# Clonidine Treatment Delays Postnatal Motor Development and Blocks Short-Term Memory in Young Mice

**DOI:** 10.1371/journal.pone.0114869

**Published:** 2014-12-22

**Authors:** Cristina Calvino-Núñez, Eduardo Domínguez-del-Toro

**Affiliations:** División de Neurociencias, Universidad Pablo de Olavide, Sevilla, Spain; Hospital Nacional de Parapléjicos - SESCAM, Spain

## Abstract

During the development of the nervous system, the perinatal period is particularly sensitive as neuronal connections are still forming in the brain of the neonate. Alpha2-adrenergic receptors are overexpressed temporarily in proliferative zones in the developing brain, reaching a peak during the first postnatal week of life. Both stimulation and blocking of these receptors during this period alter the development of neural circuits, affecting synaptic connectivity and neuronal responses. They even affect motor and cognitive skills later on in the adult. It's especially important to look for the early neurological consequences resulting from such modifications, because they may go unnoticed. The main objective of the present study has been to reaffirm the importance of the maturation of alpha-adrenergic system in mice, by carrying out a comprehensive examination of motor, behavioral and cognitive effects in neonates, during early postnatal development, following chronic administration of the drug Clonidine, an alpha2 adrenergic system agonist. Our study shows that mice treated postnatally with clonidine present a temporal delay in the appearance of developmental markers, a slow execution of vestibular reflexes during first postnatal week of life and a blockade of the short term memory in the novel object recognition task. Shortly after the treatment the startle response is hyperreactive.

## Introduction

In many experimental animal models, neonates are born immature, and the developmental stage of the brain corresponds closely to that of the newborn human. During the animal's development, drugs can act on neurotransmitter or endocrine systems, inducing brief changes during this period of fast brain growth, which is the perinatal period [1]–[2]. Especially important are the neurological consequences resulting from such situations, because at first they may go unnoticed.

During postnatal development, a critical period for the neuroendocrine and behavioral development, α_2_-adrenergic receptors raise their maximum expression values at the level of the brainstem [3]–[4]), and have been proposed as possible regulators of different processes during development [5]–[6]. Neonatal manipulation of these receptors at the level of the pons has been demonstrated to have consequences in the adult [7], affecting reflex responses such as startle and pre-pulse inhibition [8].

Clonidine, an agonist of α_2_-adrenergic receptors, applied chronically, reduces neonatal noradrenaline (NA) brain levels and causes hypersensitivity to NA at the CA1 cells in the hippocampus, permanently affecting plasticity and epileptogenic kindling in adults [9]. Chronic clonidine treatment also affects the development of REM sleep in neonatal rats and has been broadly used in REM sleep deprivation studies [1], [10]. As adults, these treated rats appeared hyperactive and hyperanxious, and had a diminished sexual behavior, with sleep disorders and general cortical-size reduction.

Eight-day-old neonates show reduced levels of α_2_-adrenergic receptors in the cortex as compared with the brainstem. Thus, clonidine may be less active on the cortical region. However, the fact that clonidine increases the expression level of the mRNA of the apoptotic enzyme caspase-3 as measured by rt-PCR, intensifying DNA fragmentation, suggests that clonidine is facilitating neuronal death during postnatal cerebral development [11].

Most of the experiments carried out in experimental models have been performed on rats and focused on the long-lasting effects in the adult after chronic neonatal clonidine treatment. However, it must be borne in mind that the adult phenotype can be affected by a deficit in one or more functional domains during neurodevelopment. In this sense, for the first postnatal stages, only the sleep-wake cycle, as well as general exploratory activity, has been dealt with, and again in rats.

Under the assumption that clonidine treatment in immature mice affects the maturation of the noradrenergic system and its functional rostral projections, the general aim of this project was to carry out a comprehensive examination of postnatal development of the motor activities and behaviors in these treated mice. We investigated the consequences of a chronic clonidine treatment on the daily neurobehavioral development of sensory and motor responses during the first three weeks of life, by using a complete test battery.

## Methods

### 1. Experimental Subjects (Ethics Statement)

Experiments were carried out on CD-1 mouse pups aged between 1 and 30 days after birth (named P1 and P30, respectively). We used 12 litters, obtained by mating WT males and females (from Harlan laboratories). Animals were housed in standard boxes (together with their mother until they were weaned off at P21) and kept on a 12 h light/dark cycle with constant ambient temperature (22±1°C) and humidity (60±5%). Food and water were available *ad libitum*. All measurements were made between 8.00 am and 14.00 pm. Six of these litters (n = 40 neonates) were treated daily with clonidine (Sigma) by subcutaneous administration of the drug (dose of 35 µg/kg) dissolved in saline, applied 30 min before undergoing the test battery. Treatment started at P1 and finishes at P22 (weaning). Treated neonates (CLO) were apparently normal, with no mortality observed during the time of analysis. As controls, the other six litters (n = 40 neonates) were treated just with saline. Severe stress due to daily handling and injections was minimized as much as possible. Pharmacological and behavioral studies were performed in accordance with the guidelines of the European Union Council (2003/65/EU) and current Spanish regulations (BOE 252/34367-91, 2005) for the use of laboratory animals in chronic experiments. Experiments were also approved by the Ethics Committee for Animal Care and Handling of the Pablo de Olavide University.

### 2. Postnatal test battery

We completed the standard Fox battery described elsewhere [12], which provides an assessment of development throughout the neonatal period. On the second postnatal day (P1), and then daily, neonatal mice were individually weighed and examined for developmental milestones and complex motor behaviors, and placed again with their mother. In addition to the “day of first performance” for each behavior, during the following days, the time required to perform each test was recorded. All timed responses were limited to a maximum of 30 sec. A non-responding animal was scored at 30 sec [13]. The behaviors evaluated were the following:

#### 2.1. Suction

Suction activity counted the number of jaw openings elicited by a peribuccal stimulus (small plastic catheter) during 30 sec.

#### 2.2. Surface righting

First day and time in seconds for pups placed in a supine position to return to the prone position with all four paws on the ground.

#### 2.3. Rooting

The first day that the head turned toward the side of the face being stroked with the tip of a cotton swab.

#### 2.4. Cliff aversion

The first day and time in seconds for pups positioned with forepaws and nose over the edge of a shelf to turn (90°) and begin to crawl away from the edge.

#### 2.5. Negative geotaxis

The first day and time in seconds for pups placed head down on a 30 degree ramp to turn 180 degrees and begin to crawl up the slope.

#### 2.6. Pinna detachment

The first day that both ears were open.

#### 2.7. Eye opening

The first day that both eyes were open.

#### 2.8. Ear twitch

The first day that the ear twitched after stimulation with the tip of a cotton swab.

#### 2.9. Forelimb grasping

The first day pups could remain suspended for at least 1 sec after grasping a thin rod with their forepaws, and time in seconds on the following days.

### 3. Startle response

The appearance of the reflex was measured by the presence of the animal's shaking after a sudden sound (a clap at a distance of 25 cm, louder than 100 dB). This was tested from postnatal day 7 (P7) until the day all the subjects presented the reflex. Quantification of motor responses was made using a specific apparatus (Cibertec, S.A.) with its software as in [14]. This task was carried out at P30, being the only one performed after the treatment had finished. The mouse was introduced individually into a soundproof box above a pressure sensor which detects any vibration caused by movement. A 100 ms sound was applied every 30 sec with an intensity increasing every 5 min, during a session that lasted half an hour (75, 85, 95, 105, 115 and 125 dB; 10 sounds per intensity). Before the first sound emission there was an acclimatization time of 3 min. The dependent variables analyzed were the *Response latency* (time in seconds the mouse took to react after the sound emission) and *Response area* (analyses from the curve represented by the force exerted by the mouse on the surface during the acoustic stimulus, mNew x s).

### 4. Hot-plate

P18 mice were screened by placing them on a hot-plate maintained at 52.2°C [15]. The variables measured were Sensitivity (T_1_, time in seconds until the first time the mouse licked its paws) and Pain Behavioral Response (T_2_, time in seconds until the animal reared three times leaning against the wall or jumped). Cut-off was 240 seconds.

### 5. Open field

Exploratory locomotor activity and anxiety-like behavior of mice was evaluated by placing the animal in a square open field actimeter (35×35×25 cm, Cibertec). The box has infrared rays (16×16) that measure the locomotor activity when the mouse crosses them, in both X and Y axes. Lighting and sound conditions were standardized across all session. Locomotor activity was measured for 10 minutes in P16 mice (just after eye opening) with the software MUX_XYZ16L. The dependent variables recorded included cumulative activity and percentage of time spent in the center of the field.

### 6. Novel object recognition (NOR) task

NOR training relies on a rodent's spontaneous tendency to explore objects, spending more time exploring the novel one than a familiar one [16]–[17]. P20 subjects were habituated individually to an empty exploratory box (40×25×20 cm) for 5 minutes. Immediately after habituation, a training session was conducted for 5 min by adding two equal objects (object A and A_1_), positioned in two adjacent corners, 10 cm from the walls. In a short-term retention test, 1 h after training, mice explored the open field for 5 min in the presence of one familiar (A) and one novel (B) object. In a long-term retention test, 24 h after training, mice explored the box for 5 min in the presence of the familiar object (A) and a novel one (C). The same groups of animals were submitted to NOR testing trials at 1 h and 24 h after training. NOR procedures were conducted in a softly illuminated room in order to minimize the influence of contextual information. Objects were all made of the same material, with different shapes and they had been previously tested in other mice in order to discard any kind of preference to any of them.

Exploration was defined as sniffing or touching the object with the nose and/or forepaws. Data were analyzed by calculating a recognition index for each animal, expressed as the ratio T_B_/(T_A_+T_B_) [T_A_ =  time spent exploring the familiar object A; T_B_ =  time spent exploring the novel object [18]. A recognition index of 0.5 shows no preference for either object, as it was always tested in the training session.

### 7. Statistical analysis

All values are reported as means ± S.E.M. Comparisons between groups were made by a t- Student test, and for the differences between sessions were analyzed with repeated measures ANOVA, using SPSS 18 software. In all comparisons, p<0.05 was considered to indicate statistical significance.

## Results

### Effect of clonidine treatment on the appearance of earliest reflexes and developmental markers

In order to study the postnatal effect of clonidine treatment on the psychomotor development in mice, we carried out a serie of tests, much of which are collectively known as the Fox battery. These tests were performed during the first two weeks of life in pups treated daily with clonidine and were compared with results in control pups. No effect of the treatment was observed on either survival or postnatal weight evolution (F_(13,60)_ = 2.638; p = 0.109). Postnatal suction activity also appeared normal (at P5, 35.69±1.27 jaw opening in control vs. 32.58±1.30 jaw opening in CLO, p>0.092). The only significant differences were observed at P1 (21.64±1.64 jaw opening in Control vs 32.58±1.10 jaw opening in CLO; p<0.001 ). We tested behavior during the following week of life in additional tests.

Data from the neurobehavioral battery of tests are summarized in Table 1, which shows the first day of performance of the tasks for the neonates of the two experimental groups (shown as means ± S.E.M.). It is noteworthy that the possible sedative or numbing effect of the treatment did not inhibit the performance of any of the tasks, but, as can be seen, the implementation of most of them in the clonidine-treated group was delayed with respect to the control group.

**Table 1:** Summary table representing the first day of implementation (Means ± S.E.M.) of the different tests for the neonates from the two experimental groups.

TEST	CONTROL	CLO	P VALUE
Surface righting	1.19±0.11 (n = 35)	2.11±0.11 (n = 35)	<0.001 *
Cliff aversion	1.85±0.13 (n = 35)	2.74±0.11 (n = 35)	<0.001 *
Negative geotaxis	3.63±0.28 (n = 45)	4.07±0.21 (n = 45)	0.203
Forelimb grasping	7.23±0.07 (n = 40)	7.03±0.04 (n = 40)	0.276
Rooting	3.23±0.15 (n = 26)	3.88±0.26 (n = 26)	0.035 *
Pinna detachment	4.13±0.09 (n = 45)	4.71±0.10 (n = 45)	<0.001 *
Startle response	8.20±0.07 (n = 35)	10.00±0.40 (n = 35)	<0.001 *
Ear twitch	5.11±0.18 (n = 40)	10.28±0.12 (n = 40)	<0.001 *
Eye opening	14.21±0.08 (n = 24)	15.25±0.11 (n = 24)	<0.001 *

The delay in the developmental landmarks was significant. Pinna detachment occurred at day 4.13±0.09 in control mice, earlier than in clonidine-treated mice (day 4.71±0.10, p<0.001). Eye opening also occurred one day later in clonidine-treated mice (day 14.21±0.08 in control group vs. day 15.25±0.11 in CLO group, p<0.001).

Some reflexes elicited by stimulation with the tip of a cotton swab also appeared later (rooting: day 3.23±0. 15 in control vs. day 3.88±0.26 in CLO, P<0.001) (Ear twitch: day 7.38±0.35 in control vs. day 9.37±0.32 in CLO, p<0.05).

Forelimb grasping appeared the first time with no significant differences between the two groups (day 7.23±0.07 in control vs. day 7.03±0.17 in CLO, p = 0.276).

### Postnatal clonidine treatment effects on the development of the vestibular system

**Figure 1:**
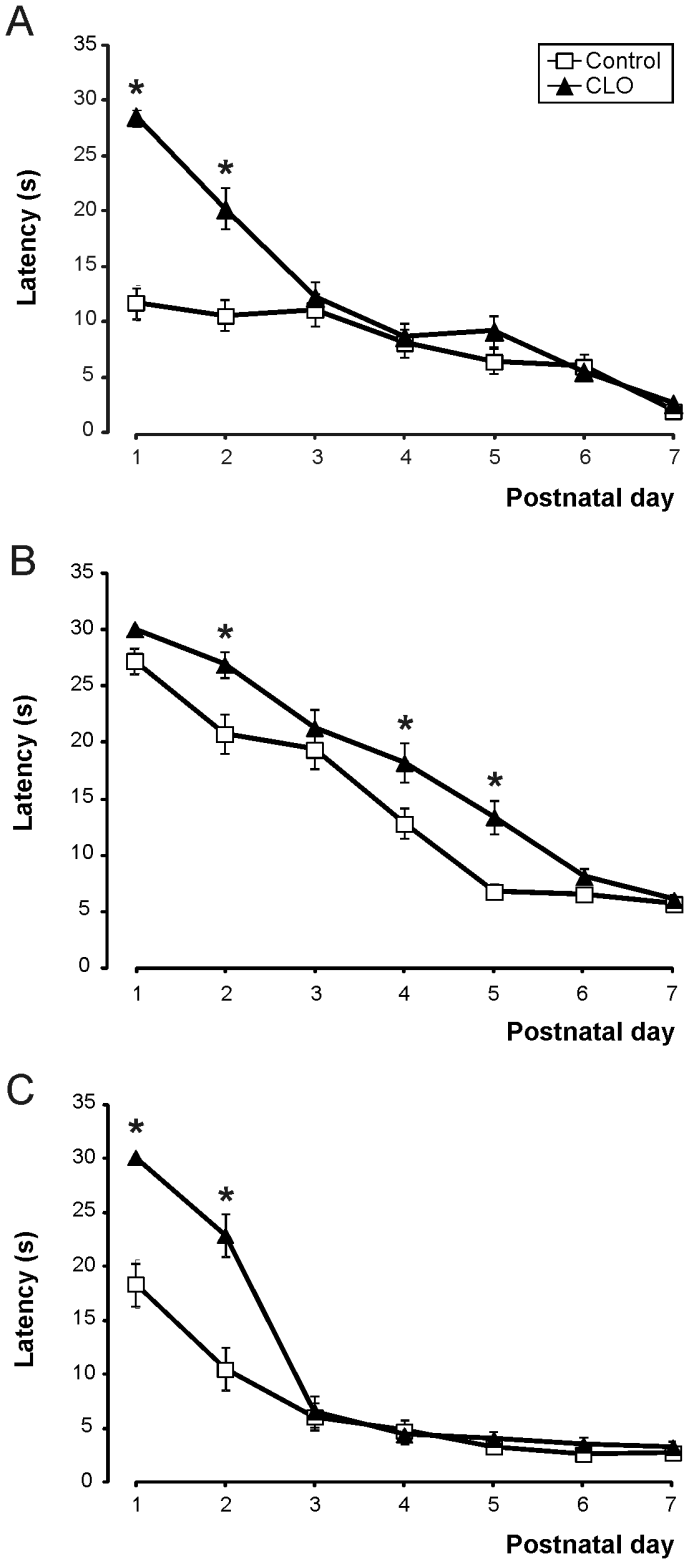
Clonidine treatment delays postnatal development and motor performance in different vestibular tests. ***A***, Righting reflex. Postnatal evolution of latencies (in seconds) required in modifying position to a final prone position. Number of mice N = 35control:35CLO. ***B***, Negative geotaxis. Postnatal evolution of latencies (in seconds) required in modifying by 180 degrees the orientation on a slope. N = 45∶45. ***C***, Cliff aversion. Postnatal evolution of latencies (in seconds) required in escaping from a cliff. N = 35∶35. * significant differences between control and clonidine (CLO) -treated mice, when p<0.05.

Some of the tests performed involve a spatial reorientation of the neonate. The surface righting, as seen in Table 1, appeared almost one day later in clonidine-treated mice (day 1.19±0.11 in control vs. day 2.11±0.11 in CLO, p<0,001). With regard to the time spent for the movement, from the first moment, together with the mentioned delay, there was normally a reduction in the time required. Control mice moved from 11.79±1.60 seconds the first day to 1.98±0.38 seconds the seventh day. Clonidine-treated mice evolved in a similar way, with longer times (28.62±0.61 seconds the first day, to 2.66±0.26 seconds the seventh day) (F_(6,63)_ = 36.716; p<0.001) (see Fig. 1A).

Negative geotaxis appeared the same day in clonidine-treated mice, and in control ones (day 3.63±0.28 in control vs. day 4.07±0.21 in CLO, p = 0.203, Table 1). As seen in fig. 1B, a delay appears in the evolution of the reflex, with a trend towards a reduction in time during the following days until a stable time was reached on the sixth day (seventh in the CLO group) (F_(6,83)_ = 12.076; p = 0.001).

The cliff aversion test also prompted mice to change position, escaping away from the edge. As we can see in Table 1, clonidine-treated mice made the movement for the first time with some delay as compared with control mice (day 1.85±0.13 in control vs. day 2.74±0.11 in CLO, p<0.001). The delay was mirrored in the time required to complete the task, which continued the following days (see Fig. 1C). The second day, for example, control mice made the movement in 11.03±1.96 seconds, while CLO mice needed 23.03±1.90 seconds (p<0.001 ). F_(6,63)_ = 18.548; p<0.001.

### Clonidine treatment increases the startle response intensity

The Startle response appeared the first time with significant differences between the two groups (day 8.20±0.07 in control group vs. day 10.00±0.40 in CLO group, p<0.001, see table 1). At P22 we analyzed and quantified the variables *Response latency* and *Response area*.

*Response latency* is the time in seconds the mouse took to react after the sound emission. The most notable element of the results is that at low intensities (75 dB) clonidine-treated mice had a greater *Response latency* compared with control mice (47.17±6.42 ms and 28.06±3.87 ms, p = 0.043). The evolution of the *Response latency* for the higher intensities is almost parallel in the two groups of mice, with a general trend of a decrease in *Response latency* with increasing intensity of sound.

**Figure 2:**
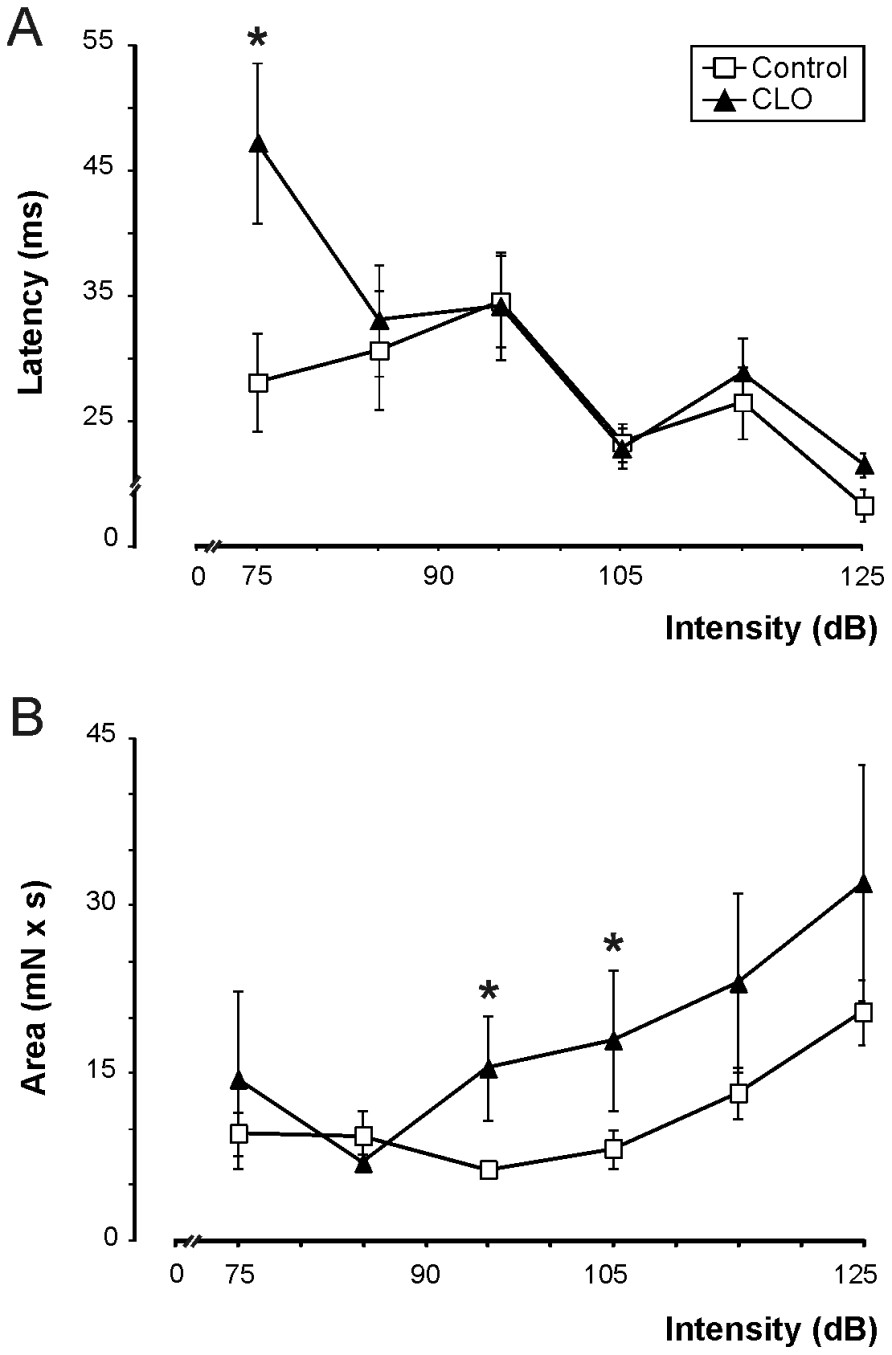
Effect of clonidine treatment on startle reflex parameters at different sound intensities. ***A***, Latencies (in ms) until the appearance of the motor response reduce as intensity increases (75, 85, 95, 105, 115 and 125 dB), mainly at higher intensities. No differences are observed between control and clonidine-treated mice, apart from the case of the lowest intensity (75 dB). ***B***, Area (in mN x s) of the increase in startle response with the intensity of the sound. Clonidine-treated mice show larger areas at intensities above 95 dB than control mice. N = 35∶35. * significant differences between control and clonidine (CLO) -treated mice, when p<0.05.

The other analyzed variable is *Response area*, obtained from the curve represented by the force exerted by the mouse on the surface during the acoustic stimulus (mNew x s). For this variable, we can see that from 95 dB on, the *Response area* for clonidine-treated mice is much greater than for controls, and its pattern remains almost parallel. In both groups of mice, *Response area* tends to increase with the sound intensity. At 95 dB and 105 dB intensities, the results reached statistical significance (p = 0.022 and p = 0.008) (Fig. 2).

### Postnatal clonidine treatment reduces escape attempt latencies after pain stimulus

**Figure 3:**
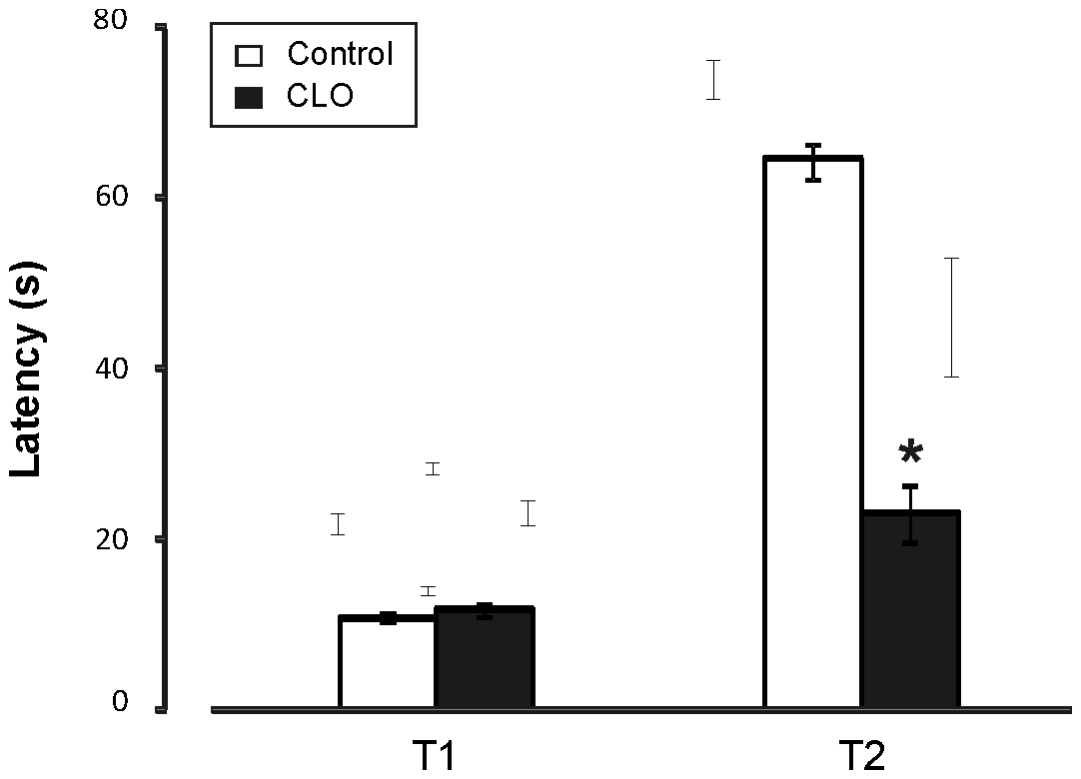
Nociceptive and behavioral responses in the hot-plate test after clonidine treatment. The first latency (T1, in seconds) shows the sensibility of clonidine-treated mice compared with control mice. The second latency (T2) shows the behavioral response as the third escape attempt, greater in control mice. N = 21∶21. * significant differences between wild-type (WT) and clonidine (CLO) -treated mice, when p<0.05.

For pain sensitivity, there are no differences between the values obtained for the clonidine-treated group and for the control one (11.08±0.54 controls vs. 11.74±0.80 for CLO, p = 0.501). For Behavioral Response to pain, the control group presented higher latencies than the clonidine-treated group (64.51±2.08 in controls vs. 23.11±3.48 in CLO, p<0.001) (Fig. 3).

### Postnatal clonidine treatment reduces motor exploratory activity

This task evaluates exploratory capacity or motor activity of mice after opening of the eyes. Measurement of activity is represented as an accumulation over time.

**Figure 4:**
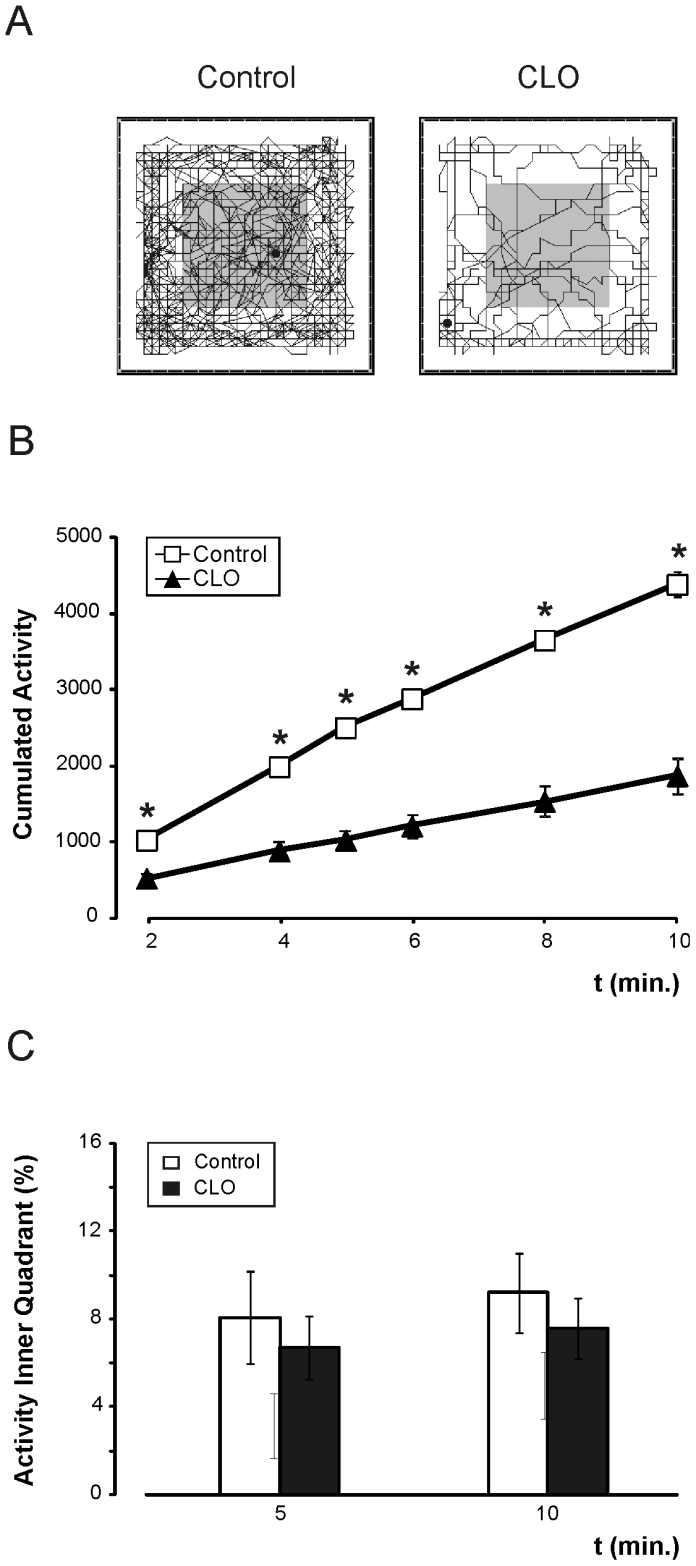
Reduction of spontaneous exploratory activity in the open field test after clonidine treatment. ***A***, Schematic representation (obtained from MUX_XYZ16L software) of the accumulated movement performed during 10 minutes for control (left) and clonidine-treated (CLO, right) mice on the box surface. ***B***, Evolution of the accumulated activity during the 10-minute test, showing the maintained reduced activity of clonidine-treated mice. ***C***, Percentage of total time spent in the inner quadrant in 5 and 10 inutes for control group (white bar) and CLO group (black bar). N = 21∶21.

At 5 min., exploratory activity of the control group was greater than that of the clonidine-treated group (2156.57±68.52 units vs. 914.29±109.25 xy ray cuts, p<0.001). At 10 minutes, the exploratory capacity remained higher for the control group (3735.81±134.37 xy ray cuts) than for the clonidine-treated group (1625.14±195.25 xy ray cuts, p<0.001) (Fig. 4A-B). In general, exploratory activity was greater for the Control mice than for the clonidine-treated group (F_(5,36)_ = 91.442; p<0.001).

As a possible anxiety index, activity in the inner quadrant was measured. There is no difference between experimental groups (at 10 min, 9.20±1.81 in controls vs. 7.61±1.37, F_(1,40)_ = 0.548; p = 0.464) (Fig. 4C).

### Postnatal clonidine treatment affects memory development and reduces Recognition Index in the novel object recognition task

**Figure 5:**
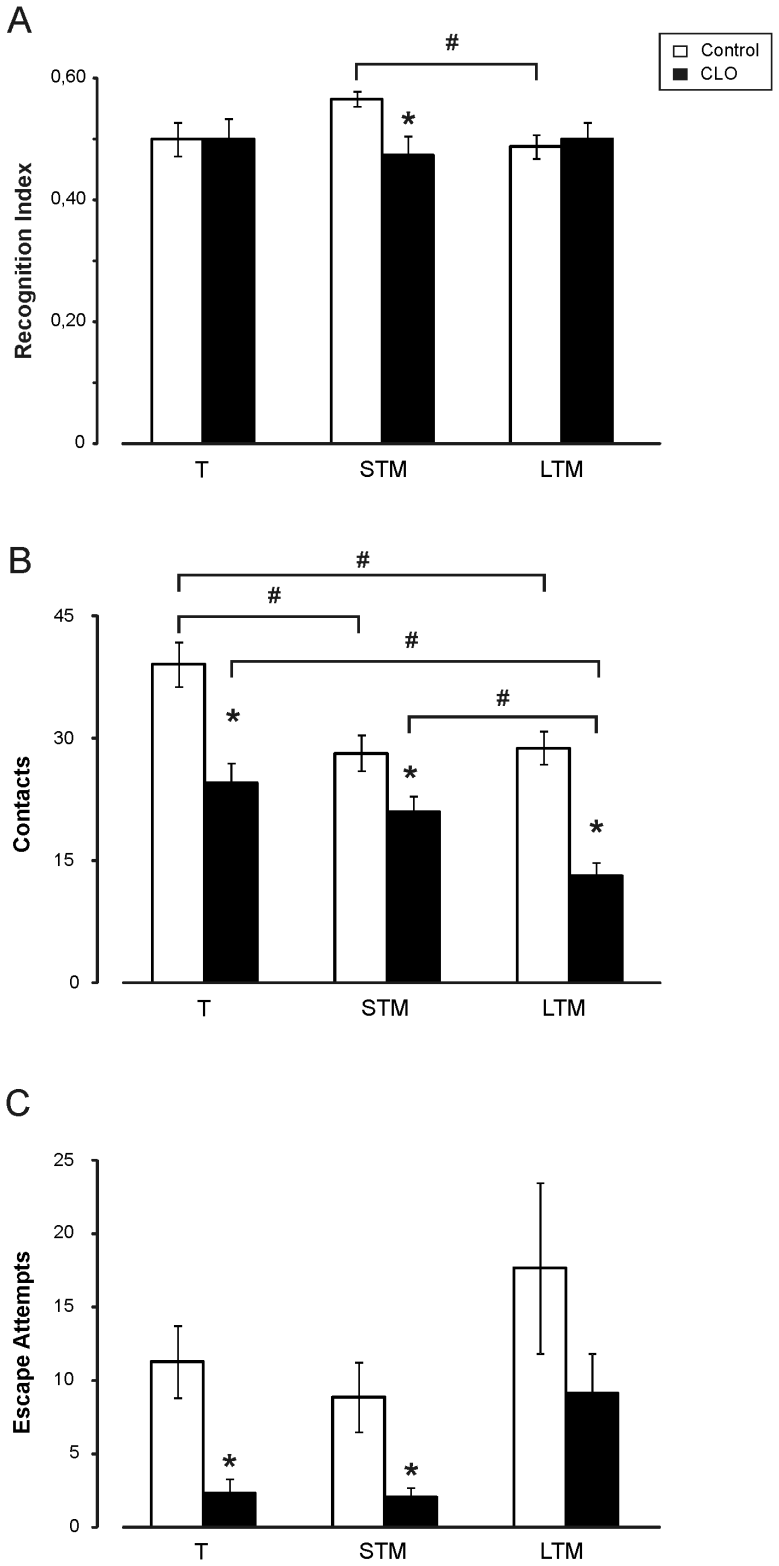
Effects of clonidine treatment during novel object recognition (NOR) memory test in mice. ***A***, Recognition Index. The CLO-treated group (CLO) presents no increase in the Recognition Index [T_B_/(T_A_+T_B_)] for the short-term retention test (STM) and the long-term one (LTM), compared with the increase normally observed in control subjects. ***B***, Total contacts with both objects. The CLO-treated group presents, during the training session (T), a significantly reduced object exploration. There is no reduction of object exploration in the short-term memory test for this group, but there is for the long-term memory test. ***C***, Escape attempt. During training and short-term memory tests, the CLO-treated group presents almost no tendency to escape as compared with controls. This activity is increased in the long-term memory test, but results are significantly reduced as compared with the control group. Results are presented as means±S.E.M. The statistically significant difference between the two groups is represented by the symbol *, while the symbol # is used to compare the different tests—short- and long-term retention—with training. N = 40∶40.

Using the novel object recognition (NOR) task, we could evaluate short-term (1 h) and long-term (24 h) memory of young mice when they are exposed to new and familiar objects. In this task, for the control group the Recognition Index increased to 0.57±0.01 in the short-term retention test, while the clonidine-treated group kept the same value as in training (0.48±0.03), indicating that the clonidine-treated group had no preference for either object (p = 0.002). At 24 h the indices remained at the same values than at the training test (0.49±0.02 vs. 0.50±0.03; p = 0.702) (Fig. 5A).

In this task we also measured the total contacts with the objects. The clonidine-treated group made fewer contacts than the control group for the three sessions (F_(2,77)_ = 25.845; p<0.001) (Fig. 5B).

The last variable measured in this task was escape attempts. The clonidine-treated group presented fewer escape attempts in all the sessions. In the training session, for example, the control group attempted to escape 11.30±2.48 times, compared with 2.33±0.99 times for the clonidine-treated group. (F_(2,77)_ = 6.322; p = 0.014) (Fig. 5C).

## Discussion

The first assumption to be made before starting to analyze our results is that the chronic postnatal clonidine treatment was effective. This was borne out by the fact that chronic treatment with clonidine had no effect on newborn survival and that differences obtained when comparing the two experimental groups were usually significant. In the present study we used a daily dose of 35 µg/kg. Previous studies used postnatal clonidine treatment in rat REM sleep deprivation models applying higher doses of 50 µg/kg, 100 µg/kg or 50+50 (morning/evening) µg/kg [1], [10]. The same dose used in the present study has been tested on startle responses in adult mice in [19].

An overall view of the results in all the tests carried out and the subsequent analyses performed demonstrates that in the majority of the cases, clonidine-treated mice present a temporal delay in the appearance of developmental markers, as well as in the performance of behavioral tests. This argues also in favor of the procedure. Tests were made 30 min after the treatment, and it's well established a peak of clonidine acute effect on neonatal rodent locomotion at this time point [20]. All of these animals showed significant differences when compared with control mice, except in the appearance of the startle response and the suspension test (forelimb grasping) which started at similar ages but showed differences in the execution times on the following days.

### Involvement of noradrenaline in postnatal development

With regard to the studies on physiological development and its markers, we found that mice treated with clonidine show a delay in pinna detachment and in eye opening. Although there is no effect of the treatment on feeding behavior and postnatal growth, our results agree with those from certain other studies in which manipulations causing postnatal stress in rodents also delayed these developmental markers [21]–[22]. We cannot rule out the possibility of a local vascular effect due to the antihypertensive effect of clonidine [23], which could modify blood supply to the skin, thereby affecting the normal development of these markers, as has been recently demonstrated for the hormone vasopressin [24].

From the first two reflexes appearing in newborns (rooting and suction reflexes), we can reiterate that animals treated with clonidine show a slower development compared with control animals, which presented values similar to that published previously [13]. It is surprising that the rooting reflex appears so late, as this reflex has the function of allowing the pup to find the mother's nipple, but we could argue that other stimuli as mother's odor can help the pup during these first days. Both reflexes are produced after sensitivity stimulation of the peri-oral region, innervated mainly by the trigeminal nerve. One efferent projection from the Locus coeruleus (LC) arrives at the trigeminal nucleus [25], so it can be expected that clonidine treatment, affecting the firing rate of LC neurons, secondarily affects these reflexes. In fact, in newborn mice suffering from deletions in the trigeminal regions, a severe reduction of the suction behavior is observed [26].

Analyzing suction behavior by the number of mastications, we demonstrated that mice treated with clonidine started the experiment with a similar number of mastications but, as the days passed, these diminished significantly. Involving the participation of the trigeminal nuclei, rhythmic movement has been shown to be affected by developmental modifications in the pontine regions [26]. Studies performed in rats after postnatal treatment with clonidine demonstrated neuronal death in the rostral part of the brainstem, the region expressing the greatest amount of adrenergic α_2_ receptors [27].

With reference to pinna stimulation, if this reflex response is related with the day of pinna detachment, which is occurring with some delay, it is logical to assume that the reflex will be delayed, as it happens.

In all the tests carried out involving the vestibular system, clonidine-treated mice showed a delay in the execution of the movement (reflex response) compared with controls, with significant differences, apart from *forelimb grasping* (probably the test that has least relationship with this system, but more with the spinal-motor system).

We can understand that clonidine interferes with the development in neonates of those brainstem centers regulating this balance system. While we found a delay for the first day on which treated neonates performed the tests, we also found that the latencies for completing them were greater, something very evident in the *righting reflex*. There are few data related to this in adults [28], with a loss of righting reflex after α_2_ agonist administration, although we must separate the sedative effect [29]–[30] from that affecting the reflex movement directly. In our neonates, we did not observe a loss of the righting reflex, but—as mentioned—a delay in its execution. We did not observe any hyperactivity during the first postnatal week, even if clonidine has been shown to induce motor activity of rat P6 pups [31]. We must also bear in mind that clonidine treatment has been associated with neuronal death in the rostral brainstem, and this may affect related regions or make the whole system function slower. An anatomical analysis would be necessary in order to verify this. There are precedents in which neonatal manipulation of α_2_ receptors at the pontine level caused functional consequences in the adult, affecting reflex responses such as the startle reflex and pre-pulse inhibition [7]. These studies suggest that the interference with noradrenergic neurotransmission to cortical regions, in this case potentiating them, affects the performance of these reflex responses. This could explain how our treatment, diminishing the neurotransmission, also affects the responses, even in young individuals.

In the *cliff aversion* test we found no great differences between the two experimental groups. While the vestibular component of the response is not so great, the anxiety is. Our results contrast with those obtained by Shishkina et al. [7], in which postnatal treatment in rats with antisense for α_2_ receptors produced, in adults, a reduction of anxiety, and those from Mirmiran et al. [1], who described cases of hyperanxiety after postnatal chronic clonidine treatment in rats. We have found no evidence of anxiety in early postnatal treated mice but, as we will discuss later, we demonstrate its existence when we tested (one week later) open field activity, with a general reduction in movement, and a significantly reduced percentage of exploration at the center of the chamber.

### Consequences of clonidine treatment in young mouse behaviors

The auditory system is directly involved in the generation of the startle reflex. We used two approaches to investigate this response. The first, directly related to the development of the system, evaluated the appearance of the motor response after the sudden sound presentation. Clonidine-treated mice showed no differences when compared with control mice, so, even if the detachment of the pinna and its movement after tactile stimulations are delayed in these treated mice, the functional development of the auditory system and the coordination with skeletal movement seems to be intact (or not affected).

In the second approach we quantified different parameters of the motor response several days after the treatment had ended. Statistical differences appeared. Analyzing the latency, only when low-intensity stimuli were applied did clonidine-treated animals show a considerable delay compared with controls. Differences are more impressive if we study the area of the response activity on the platform. In this case, areas are greater in treated mice at higher intensities. In previous studies interfering with α_2_ receptor postnatal expression in the brainstem, a reduction of the startle area was observed [8], indicating that postnatal maturation of α_2_ adrenergic signaling (and LC neuronal activity) affects later startle reflexes. In our case, neuronal death at the level of the pons may favor this reflex hyperactivity, probably due to the disappearance of processes inhibiting the motor response.

Clonidine is commonly used as an anesthetic, including as a local anesthetic in children [32]. Our first analysis doesn't corroborate this analgesic effect in treated mice as paw-licking latencies are the same compared with controls. Analyzing the second component of the response—that involving the behavioral response to pain—the explanation is more complex. With regard to the behavioral differences between treated and untreated mice, we could argue that this situation is somehow stressful, naturally originating anxiety in normal animals, and that it is reduced in treated mice. In this case, contrary to the ideas expressed by Mirmiran et al. [1], who proposed an increased hyperanxiety after chronic clonidine treatment in rats, clonidine-treated mice show less anxiety, or the analgesic effect of the drug reduces pain and stress, and allows the animals to explore and look for an escape faster.

It is of interest that postnatal clonidine treatment affects not only motor development but also later attention and memory capabilities. With the novel object recognition test, clonidine-treated mice appeared less effective when exploring the new object, in both the short- and long-memory tests. We could even talk about a neo-phobic effect, as the recognition index was not greater than 0.5, but lower (0.48), although non-significantly. We can observe not only that the clonidine treatment is, logically, producing mice that are less active, but also that they interact less with both objects and that they prefer the old (known/familiar) object. It is well known that lesions in the LC, which are functionally similar to our pharmacological approach to inactivating LC neurons, produce a reduction of locomotor activity as well as a reduction of exploratory activity in rats, indicating a new stimulus rejection [33]. In our case, it would be difficult to distinguish between attention and memory processes, but we have to consider the role played by clonidine in the working memory [34]. According to Roozendaal et al. [35], NA liberated in the basolateral amygdala favors memory processes, and consequently object recognition, but clonidine administration inhibits NA liberation, reducing memory abilities. The functional modification of cortical structures at the hippocampal level, when clonidine is administered chronically during postnatal development, affecting noradrenergic neurotransmission, and which may affect attention ability, has been reported [9].

In the actimeter test, done at P16 just after opening of the eyes, general exploratory locomotor activity was significantly reduced in clonidine-treated mice compared with controls. This is due to the effect of the treatment with an anesthetic component, which can slow overall animal reflexes and reduce cutting of the infrared beams. This reduced activity contrasts with studies from Mirmiran et al. [1] that show both hyperanxiety and hyperactivity in adult rats, after postnatal clonidine treatment. But agree with those performed under drug effect and showing hypomotility in rats older than 20 days [20]. Our detailed analysis of the activity of clonidine-treated mice, which were hypoactive, in the open field test, showed a non significant reduction of the percentage for exploration at the center of the chamber, thus indicating no increased anxiety.

By studying the behavior of the mice in an open field or other mazes, we could measure their anxiety levels. In these situations, mice generally prefer to be in a safe environment, such as at the side of the open-field box, although their natural curiosity causes them to explore more-adverse environments, such as the center of the open field. We were able to determine their anxiety level by the amount of time they spent exploring this adverse compartment of the apparatus—the less adventurous the mouse, the more anxious it is perceived to be. Many studies have shown a close relationship between anxiety and noradrenergic function [36]. Chronic postnatal clonidine treatment causes a reduction in the noradrenaline level in many brain regions. By increasing noradrenaline release with an administration of yohimbine (an α_2_-adrenoceptor antagonist), Tanaka et al. [37] obtained an anxiolytic action in two behavioral tests. This may contrast with other studies showing that in some situations, clonidine, an α_2_-adrenergic agonist, reduces anxiety symptoms [38]. In the present study, clonidine also reduced anxiety in the hot-plate test. We can argue that its antinociceptive effect makes pain less stressful. The same anxiolytic-like effect following the use of various antidepressants has been proposed [39]. The evidence suggests a strong connection between 5-HT_2A_ receptors and noradrenaline neurotransmission [39]–[40]. It has been demonstrated that another serotonin receptor (5-HT_1A_) not only is involved in the establishment of normal anxiety-like behavior but also that this effect is acting during prenatal and postnatal development [41]–[42]. Interestingly, other approaches have focused on deletion of genes affecting neurodevelopmental processes and—as a result—higher cognitive functions. For example, euchromatin histone methyltransferase 1 (EHMT1) mutant mice show some delay reaching developmental milestones [43], as well as reduced activity and exploration, increased anxiety when exposed to novel environments in the open field, or object exploration [44]. The present study, interfering with the noradrenaline neurotransmission during postnatal development, shows the consequences for both early developmental markers and reflexes and early behavioral function, such as exploratory activity and memory processes. During treatment mice showed no anxiety, and hyperreactivity appears at P30, after treatment, as it has been published in rats [1]. Proposals have been made for the interference by clonidine with noradrenaline transmission in the establishment of functional circuits in cortical areas, which caused a hypersensitivity to NA at the CA1 cells in the hippocampus [10], or by yohimbine [45], which altered functional properties in the visual cortex. The increased anxiety observed in mice not during but after chronic postnatal clonidine treatment could also be linked to alteration in cortical function, as has been proposed in Emht1+/− mice [46].

### Final considerations

The main goal of our research work has been centered on supporting the concept that the maturation of the alpha noradrenergic system during early postnatal development is important for the correct development of behavioral and cognitive responses. This has been approached by chronic clonidine administration which, acting on α_2_ receptors, blocks electrical activity of the LC neurons. We conclude that clonidine-treated mice present a general delay in postnatal psychomotor development and blocking of short term memory.

Thus, although clonidine is commonly used in children as an anesthetic or in pregnant mothers as a hypertensive, the neuronal death associated to the treatment and the effects observed in activities vital for the intellectual development of the neonate, such as its sleep period [1], [11], [24] or those effects on psychomotor development observed in the present work, advise against its chronic use during this postnatal period.
